# Histological classification of canine and feline lymphoma using a modular approach based on deep learning and advanced image processing

**DOI:** 10.1038/s41598-023-46607-w

**Published:** 2023-11-09

**Authors:** Andreas Haghofer, Andrea Fuchs-Baumgartinger, Karoline Lipnik, Robert Klopfleisch, Marc Aubreville, Josef Scharinger, Herbert Weissenböck, Stephan M. Winkler, Christof A. Bertram

**Affiliations:** 1 Bioinformatics Research Group, University of Applied Sciences Upper Austria, Softwarepark 11-13, 4232 Hagenberg, Austria; 2 Department of Computer Science, Johannes Kepler University, Altenberger Straße 69, 4040 Linz, Austria; 3 Institute of Pathology, University of Veterinary Medicine Vienna, Veterinärplatz 1, 1210 Vienna, Austria; 4 Technische Hochschule Ingolstadt, Esplanade 10, 85049 Ingolstadt, Germany; 5 Institute of Computational Perception, Johannes Kepler University, Altenberger Straße 69, 4040 Linz, Austria; 6 Institute of Veterinary Pathology, Freie Univerisität Berlin, Robert-von-Ostertag-Str. 15, 14163 Berlin, Germany

**Keywords:** Lymphoma, Machine learning

## Abstract

Histopathological examination of tissue samples is essential for identifying tumor malignancy and the diagnosis of different types of tumor. In the case of lymphoma classification, nuclear size of the neoplastic lymphocytes is one of the key features to differentiate the different subtypes. Based on the combination of artificial intelligence and advanced image processing, we provide a workflow for the classification of lymphoma with regards to their nuclear size (small, intermediate, and large). As the baseline for our workflow testing, we use a Unet++ model trained on histological images of canine lymphoma with individually labeled nuclei. As an alternative to the Unet++, we also used a publicly available pre-trained and unmodified instance segmentation model called Stardist to demonstrate that our modular classification workflow can be combined with different types of segmentation models if they can provide proper nuclei segmentation. Subsequent to nuclear segmentation, we optimize algorithmic parameters for accurate classification of nuclear size using a newly derived reference size and final image classification based on a pathologists-derived ground truth. Our image classification module achieves a classification accuracy of up to 92% on canine lymphoma data. Compared to the accuracy ranging from 66.67 to 84% achieved using measurements provided by three individual pathologists, our algorithm provides a higher accuracy level and reproducible results. Our workflow also demonstrates a high transferability to feline lymphoma, as shown by its accuracy of up to 84.21%, even though our workflow was not optimized for feline lymphoma images. By determining the nuclear size distribution in tumor areas, our workflow can assist pathologists in subtyping lymphoma based on the nuclei size and potentially improve reproducibility. Our proposed approach is modular and comprehensible, thus allowing adaptation for specific tasks and increasing the users’ trust in computer-assisted image classification.

## Introduction

For automated analysis of histological images, algorithms based on artificial intelligence (particularly deep learning) have been shown to achieve exceptionally high performance as well as increased reproducibility of results^1^. The improved computational power of modern computers and the increasing capabilities of deep learning-based models have enabled these algorithms to become part of medical research and diagnostic pathology service^2^.

**Figure 1:**
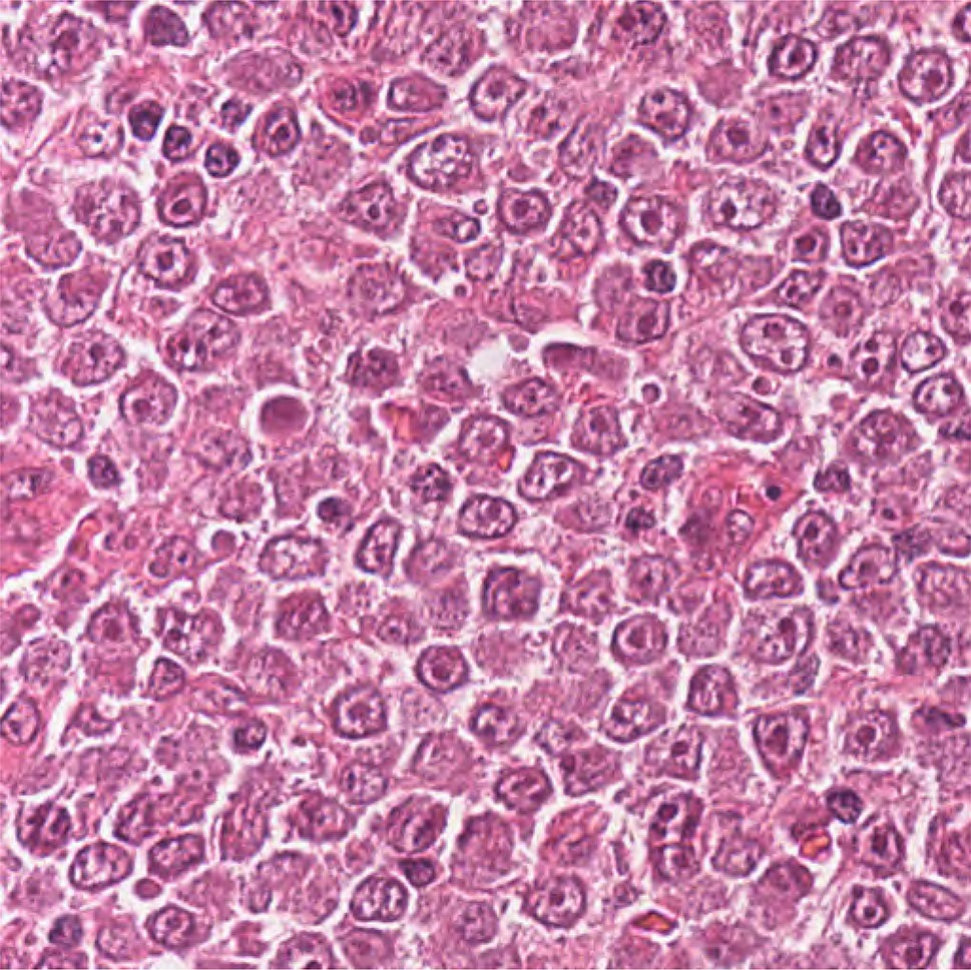
Histological slide of a canine lymphoma at high magnification with variably sized nuclei of the neoplastic lymphocytes (mostly large sized nuclei). The lack of red blood cells (size reference for lymphocyte classification) complicates size estimates. Hematoxylin and eosin stain, 40 $\times$ objective.

Lymphoma is a malignant neoplasm of the hemolymphatic system derived from lymphocytes that is common in human and veterinary medicine. Lymphoma is an umbrella term for a heterogenous group of different subtypes with highly variable biological behavior ranging from indolent to aggressive^3^. Therefore, histological classification of the different subtypes of lymphoma is necessary for the assessment of patient prognosis and decisions on appropriate treatment plans. Histological classification of lymphomas according to the WHO classification system^3^ is based on different features, including the size of the nuclei of the neoplastic lymphocytes. Nuclear size is categorized by estimating the ratio of neoplastic nuclei to the size of a red blood cell. Pathologists assign three categories: small (< 1.5 $\times$ diameter of red blood cells), intermediate (1.5–2 $\times$ diameter of red blood cells), or large (> 2 $\times$ diameter of red blood cells) nuclear size^4^. The inter- and intra-rater reproducibility of this task is not yet well studied, however, there are several aspects that may cause rater inconsistency. Besides general visual and cognitive traps for pathologists^5^, the nuclear size and shape vary between individual neoplastic lymphocytes in the same tumor, and image sections may lack an appropriate size reference (i.e., red blood cells) without changes in size and shape, as exemplified in Fig. 1. A further limitation of red blood cells as the size reference is that their size varies between species^6^.

With the routine availability of digital images (whole slide images, WSI) in the pathology workflow, accurate nuclear measurements in µm using computerized tools are theoretically possible. However, measurements of neoplastic nuclei are currently not carried out, probably due to the tedious and time-consuming nature of this task. While measurements can potentially improve reproducibility, a challenge is the lack of standardized size references in µm. Addressing the described problems, we implemented a solution for automated nuclei segmentation and subsequent image classification based on nuclear size measurements of canine and feline lymphoma using segmentation masks provided by a segmentation neural network. Although black-box models are used as a basis for our automated classification of histological images, our workflow provides reproducible and understandable results due to the insights provided into the preliminary outcomes produced during the application of the individual workflow modules. These insights provide the ability to comprehend and verify the results, which would not be possible by using classification neural networks directly trained on whole slide images.

## State of the art

In the field of digital pathology, deep learning has demonstrated its potential for detecting and classifying various types of tumors^7,8^. Collaborations between pathologists and data scientists have led to several publications highlighting the benefits of AI-supported diagnostic workflows^1,9,10^, as well as guidelines for including AI in digital pathology^11^. While these workflows can potentially save time and reduce errors, the interpretability of the diagnostic process must be improved to increase trust in these algorithms for daily use in diagnostic workflows^12^. This is majorly restricted by the use of black-box models^9^. Neural network architectures, specifically those based on convolutional neural networks, are commonly used in AI-supported histopathological diagnostics. These networks are able to quickly process large images while maintaining a high level of classification accuracy^13,14^. This is also the case for subtype classification of canine tumors^15^, which supports our strategy of relying on these models. The specific task of detecting and segmenting nuclei within histopathological images using artificial neural networks has been proven to be effective, as demonstrated in various studies^16–18^. Therefore, we did not focus our work on further comparison of different types of segmentation models based on their segmentation capabilities or further development to improve these types of models. We relied on popular neural networks for image segmentation tasks, such as the Unet^19^ and Stardist^20^. Our selected neural networks should only be considered as possible candidates for users of our modulary workflow, which could be replaced by any capable segmentation neural network. Built up by an encoder/decoder structure, Unet is a common choice for image segmentation and various adapted versions like the Unet$++$^21^, which was used for our workflow, are available. Unlike Unet, Stardist is considered an instance segmentation approach that can detect overlapping objects within an image. Both models offer precise information on the location of nuclei in the image, which serves as the foundation for our interpretable nuclei classification method.

## Material and Methods

### Image acquisition and annotation

For this study, 116 histologically confirmed canine lymphoma cases and 38 feline cases were selected from the diagnostic archive of the Institute of Pathology of the University of Veterinary Medicine Vienna. During the selection process of these cases, care was taken to include a roughly equal number of small, intermediate, and large cell lymphoma. Formalin-fixed and paraffin-embedded specimens were retrieved, and 2–3 µm sections were produced and stained with hematoxylin and eosin (HE). Out of these histological slides, 12 slides were digitized with the Aperio slide scanner (Aperio Scanscope CS2, Leica, Nussloch, Germany), and all the other slides with the 3DHISTECH slide scanner (3DHISTECH Pannoramic Scan II, Budapest, Hungary) at a magnification of 400 $\times$ with an image resolution of 0.25 µm/pixel. Within the whole slide images, a pathologist selected a representative area, and tiff files with the size of 1024 $\times$ 1024 pixels were created.

**Figure 2:**
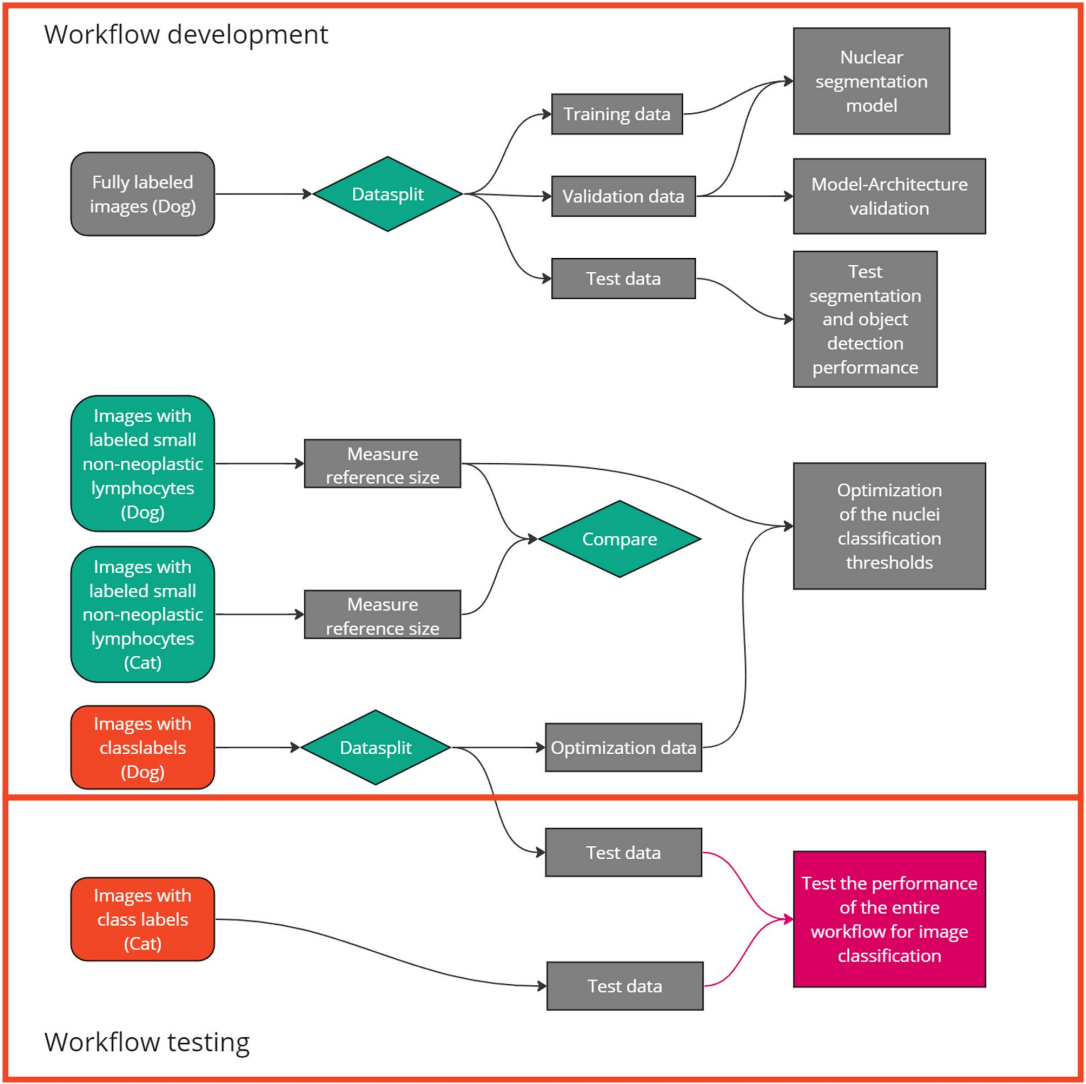
Flowchart of the experiment workflow representing the data processing for the individual workflow steps. A set of images fully labeled for neoplastic nuclei were separated into a fixed set of training, test, and validation set. Measurements of small non-neoplastic lymphocytes were used as size reference and one set of images from dogs was used for the parameter optimization. The testing of the overall lymphoma classification performance was done on independent canine and feline lymphoma images.

The individual parts of our workflow are built up by the concept of providing as much insight into the decision process for the user as possible besides the black box model used for the segmentation. Based on the separation of classification and segmentation, we used separate datasets for the individual parts of the workflow. This decision should prevent data bias during the individual steps. This separation of data can be seen in Fig. 2. The datasets were selected based on the specific objectives of each module, providing diverse information. Lymphoma images with ground truth annotations of all neoplastic nuclei were used for establishing and testing the Unet segmentation neural network model. For the subsequent image processing, we used further datasets: 1) measurements of small non-neoplastic canine and feline lymphocytes as a new reference size, 2) images from canine lymphoma classified by their overall nuclear size (consensus by three pathologists), which were used for parameter optimization. Performance evaluation of the entire workflow was done using images and global nuclear size labels of canine lymphoma and, for testing species transferability, of feline lymphoma.

#### Ground truth dataset

For the training of the Unet++ segmentation model, we used 27 images of histological samples of canine lymphoma. It was important to provide a complete label mask where all lymphoma nuclei are labeled for these samples. The ground truth labeling of all lymphocytic nuclei within these images was carried out by two pathologists using the open-source annotation software SlideRunner^22^. Using the polygon tool of the SlideRunner, the nucleus of each lymphocyte was surrounded by a thin line. In addition, the estimated size class of the nucleus (small, intermediate, large) was registered in a database. This process resulted in a total of 24.556 labeled nuclei.

The 27 samples were split into 18 training samples, four validation samples, and five test samples. During this data split, we ensured that both types of scanners were included in both the training and testing data.

To avoid overfitting during the training of our model and to reduce the impact of domain shift caused by different imaging setups, we used data augmentation techniques^23^ that involved adjusting color and contrast and applying various distortion methods, such as elastic, optical, and grid distortion, along with shifting, blurring, Gaussian noise, scaling, and rotation. Combined with cropping smaller parts of each image with a size of 512 $\times$ 512 (input size of our segmentation model), we were able to extend our dataset by a factor of ten using these techniques.

The used Stardist model (“2D_versatile_he”), was not trained using our data but instead was already pre-trained by the authors of the Stardist framework who trained it using the dataset of the “A Multi-Organ Nucleus Segmentation Challenge”^24^ as well as the dataset of a publication for nuclei segmentation in histopathological images^25^. In contrast to the commonly used fine-tuning of such pre-trained models, we used this model without any changes to test how well our workflow generalizes with different models regardless of the training dataset used.

**Figure 3:**
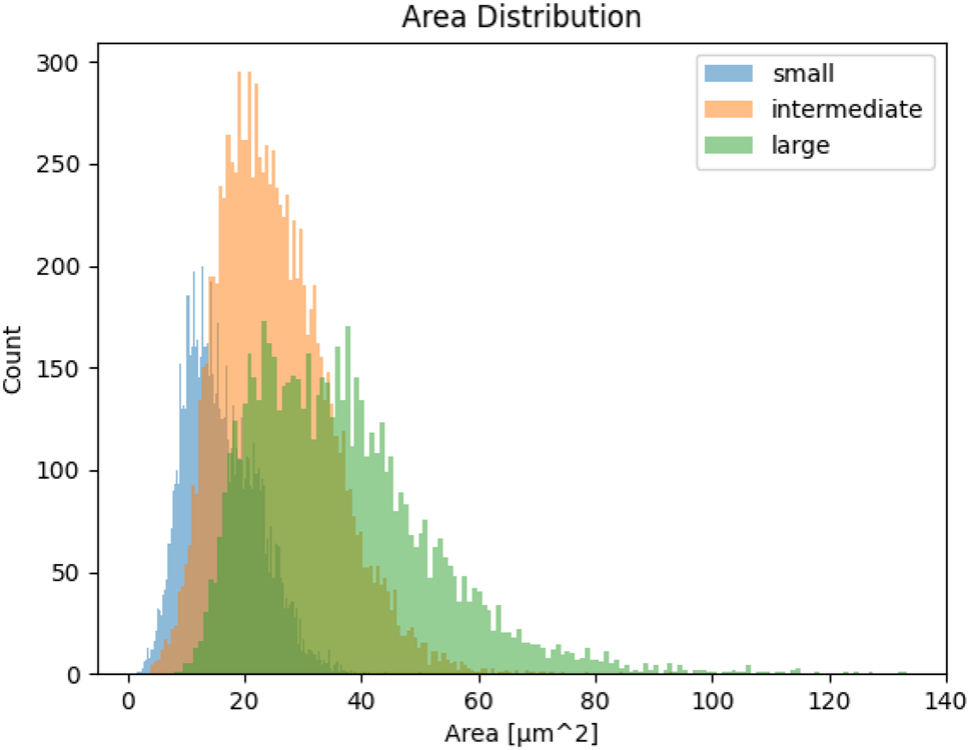
Histogram of the measured areas using the manual nuclei segmentation comparing the three label classes for the nuclear size categories estimated by the annotators (small, intermediate, large) within the training dataset (27 images). To avoid the influence of labeling errors, we have excluded objects that are less than 1µm$^{2}$ in size for this histogram.

The size estimation provided for each nucleus in the training data results in classes that overlap significantly with respect to their measured size, as shown in Fig. 3, which inhibits multiclass segmentation methods as a solution for our workflow.

#### Reference data with small non-neoplastic lymphocytes

For the selection of a valid reference size representing the fixed parameter to be used for the size comparison within our workflow, we used small non-neoplastic lymphocytes of canine and feline lymph nodes, respectively. The nuclei of 100 small non-neoplastic lymphocytes for each species were annotated as described above. The measured mean of these nuclei individually measured for each species is considered to be a representative average size of small non-neoplastic lymphocytes as well as the lower limit of the possible size for a small lymphoma nucleus. We considered small non-neoplastic lymphocytes (5–10 µm) a more appropriate reference size than the almost equally sized red blood cells ($\sim$ 6–7 µm)^6^, as they are more commonly present in most tumor regions of lymphoma (non-neoplastic lymphocytic component) and were suspected of having a more consistent size and shape.

#### Dataset for parameter optimization and workflow testing

We used 89 images of canine lymphoma and 38 of feline lymphoma, all taken from different whole slide images that were not used in the previous datasets. The canine dataset was split for parameter optimization (N = 64) and workflow testing (N = 25), while feline cases were exclusively used for workflow testing. For each image, three pathologists estimated the nuclear size category, and a label for the entire image based on the consensus of all three pathologists was created. Furthermore, the three pathologists each annotated 20 neoplastic nuclei (resulting in 60 annotations per image) for the optimization split and 10 neoplastic nuclei (resulting in 30 annotations per image) for all test images, of which the measurement was used as an alternative ground truth definition for image classification.

### Deep learning assisted image analysis workflow

**Figure 4:**
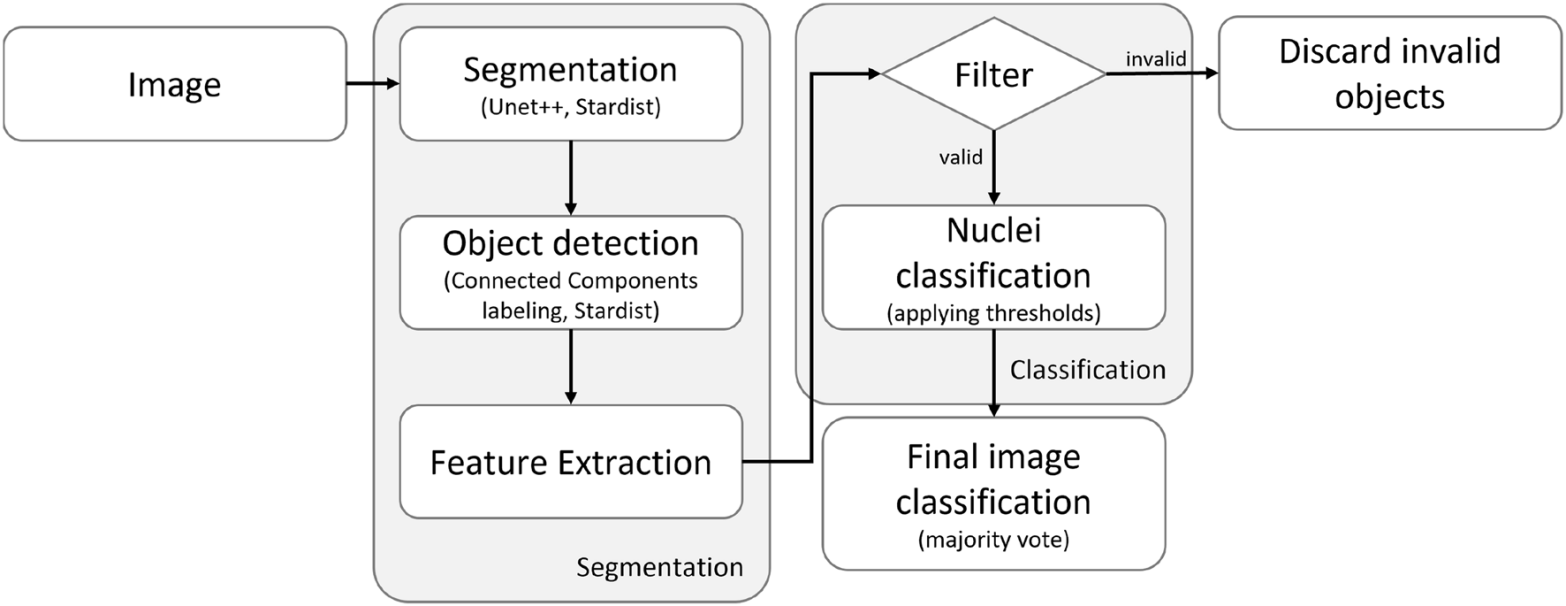
Flowchart representation of the proposed multi-step classification workflow where the initial RGB image is processed using a segmentation neural network. The resulting binary mask is used to identify and measure the individual nuclei for feature extraction. Some neural networks, like Stardist, could also provide this identification step out of the box, if it is capable of instance segmentation. After excluding all invalid objects using a filter mechanism, all remaining nuclei are classified based on the previous measurements within the feature extraction step. Using a majority vote, the most common class of nuclei also represents the final classification of the whole image.

Using an Unet^19^ architecture for the segmentation of the nuclei based on the initial RGB image, all individual nuclei are detected in the following using connected components labelling (provided by the OpenCV framework^26^). This step can be combined by using an instance segmentation algorithm like Stardist^20^, which we also included in our workflow test. Both workflow options are followed by filtering all invalid objects based on their shape. After the classification of each individual remaining object, our workflow provides a final classification for the entire image as well as the information on which objects are excluded due to the filter or the classification step, as well as the statistical distribution of the three classes. This approach delivers reproducible and understandable information in addition to the actual classification. Additionally, it is possible to adjust our workflow for other types of segmentation models and also other types of features to be extracted out of the nuclei by adjusting the individual modules.

In the following sections, we describe the individual components of our workflow as shown in Fig. 4.

#### Segmentation and detection of individual lymphoma nuclei

For our workflow, we used two different types of segmentation neural networks in order to highlight the modularity of our workflow.

Our first model is based on an Unet^19^ architecture and was specifically trained on our lymphoma samples sourced from dogs. Combined with advanced image processing used for the actual instance detection, this semantic segmentation model provided the best results on our dog data, despite its lack of detecting individual objects, as we described in the results section.

We are using an advanced version of the original Unet^19^ called Unet$++$^21^ provided by the segmentation models package for Pytorch^27^ with a regnety_120 backbone^28^. We trained this model using the Pytorch lightning^29^ framework. The training of our model was executed for 1500 epochs, and the best model of these epochs based on the validation results was selected as the final model. The modeling quality is measured using the Dice Similarity Coefficient (Dice score), where the resulting binary segmentation mask is compared with the actual label mask.

The second model represents a state-of-the-art instance segmentation model named Stardist^20^. Stardist is a deep learning framework based on Tensorflow^30^ for precise object detection and segmentation in 2D and 3D images, which already provides a pre-trained model (“2D_versatile_he”) for nuclei segmentation in histological images. This model was selected due to its generalized nuclei detection capability as well as its ability to provide the demanded instance segmentation for the following classification. It should also be able to provide this information on data sourced from different types of animals, which we test in the results section.

As shown in the segmentation result depicted in Fig. 8, the Unet++ processed the initial RGB image and generated a binary representation of the nuclei within the image. In this binary representation, all nuclei are highlighted with a pixel value of one, appearing as white objects, while the background is represented by zeros, appearing as a dark background. By applying the connected components labeling provided by the OpenCV framework^26^ on these binary images, the algorithm is able to accurately identify each individual area that was not marked with a zero pixel value. This enables the separation of each object within the binary images and the assignment of a unique identification number to each individual object. By combining the Unet++ architecture with the connected components labeling, our segmentation method provides similar information as instance segmentation algorithms like the presented Stardist without the ability to detect overlapping objects.

It is important to note that our workflow is designed to be flexible and interchangeable. This means that instead of using Unet++ and Stardist, you could also use other models if preferred. Since our primary focus was on classification based on the resulting segmentation masks, we did not create new neural network architectures for tumor nucleus segmentation, instead we relied on the sophisticated neural networks.

Based on the identification result provided by Stardist or the connected components labeling, the filtering algorithm as well as the actual classification could be applied as described in the following chapter.

#### Feature extraction and filtering of the detected nuclei

**Figure 5:**
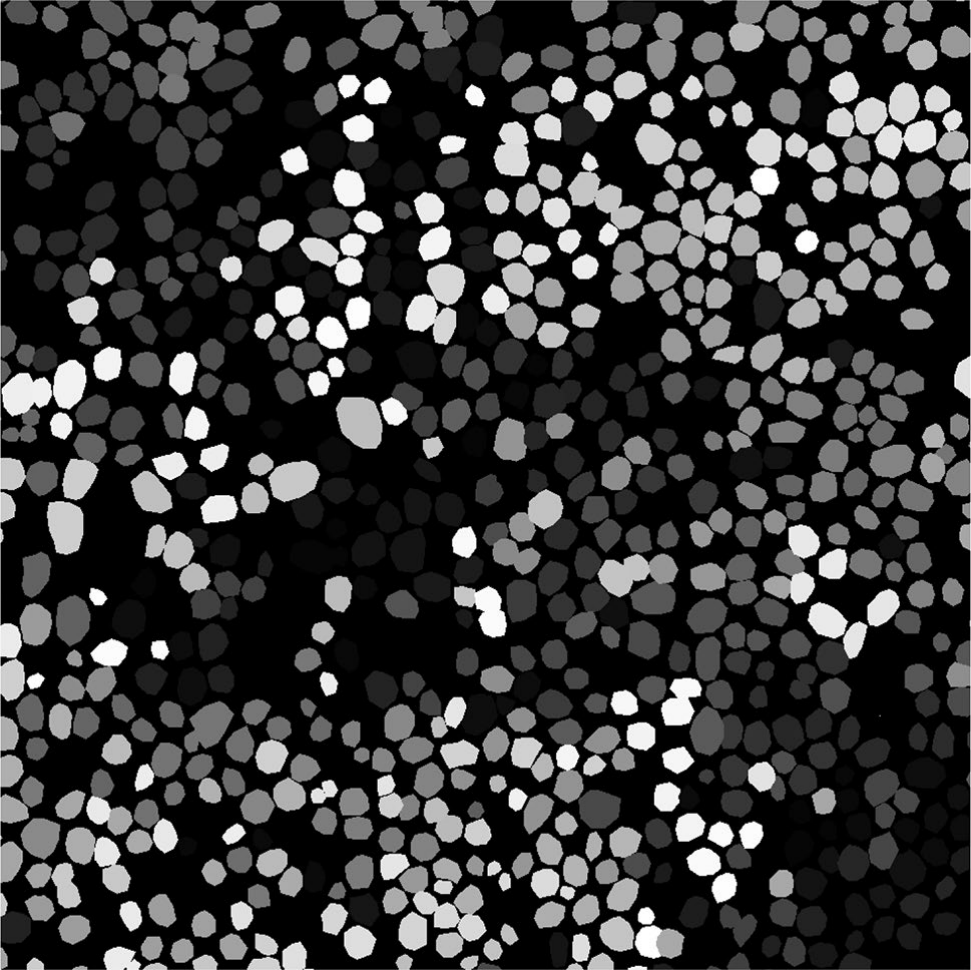
Example image of the segmented nuclei mask, where each nucleus is identified using a unique number represented by the object’s intensity value.

Following the segmentation and connected components algorithm, each identified nucleus, as shown in Fig. 5 is classified individually. While we use lower and upper limits to define the possible size of each segmented nucleus, it is still possible for closely located nuclei to be incorrectly segmented together and considered as one object. This problem is not that common using instance segmentation models like Stardist, which should result in a lower drop rate of invalid objects. To address this issue, we developed a workflow to filter out non-elliptical objects based on the hypothesis that correctly segmented lymphoma nuclei should provide a more elliptical shape than connected nuclei. Our filter mechanism involves computing the mean of two measures to calculate this so-called “circularity”, both of which are implemented using methods provided by the scikit-image framework^31^. The first measure is the ratio of the segmented area pixels to the resulting convex hull of the same area. A perfect circle should provide a ratio of one. The second measure is the ratio of the detected area to the area of a circle that uses the maximum distance of the border pixels of the detected area as the diameter. As with the first measure, a perfect circle should provide a ratio of one. To be included in the classification process, the mean of these measures must remain above 0.5 (empirically determined) for each individual nucleus. This approach ensures that only nuclei with a more elliptical shape are included in our analysis.

**Figure 6:**
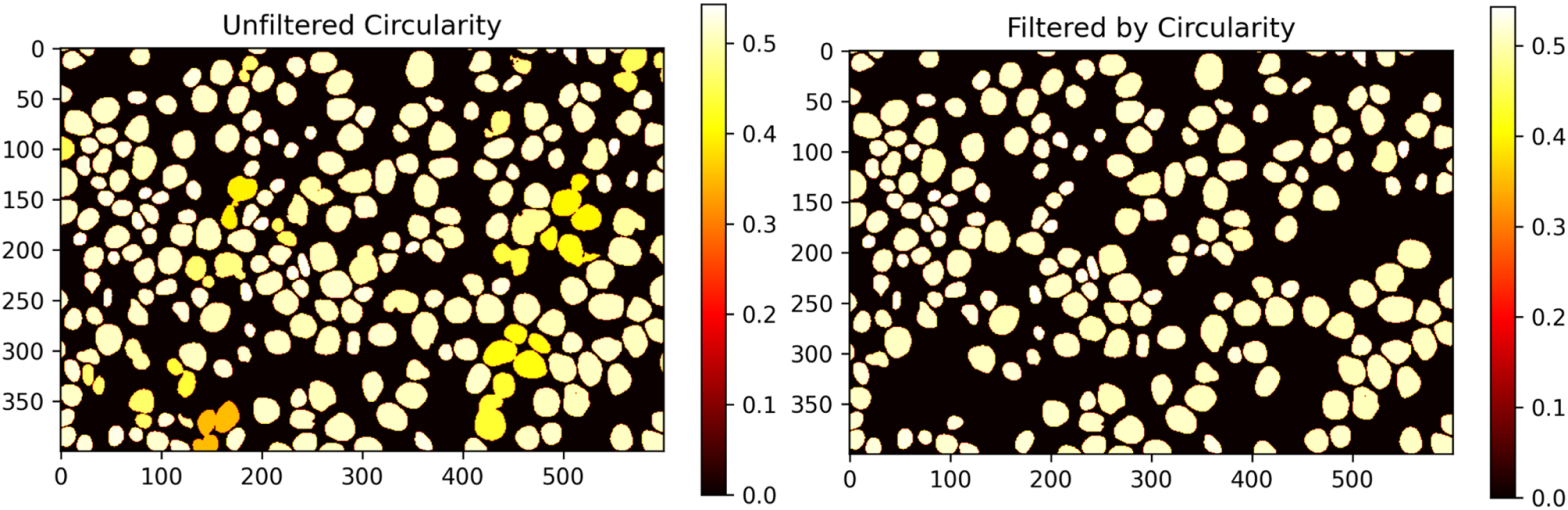
Filter mechanism comparison using the unfiltered segmentation image on the left shows some connected objects with a lower circularity score than the other non-connected objects. As shown in the filtered image on the right, these connected objects are no longer visible.

As shown in the filter example in Fig. 6, all the connected objects in the raw segmentation result are no longer visible in the filtered representation on the right due to their low circularity value.

As the reference nuclear size we calculated the mean diameter of small canine non-neoplastic lymphocytes. The overall mean diameter was 4.14 µm. This reference size provided a useful measure for classifying lymphoma nuclei in our workflow.

Based on the measured reference, finding thresholds for the actual classification is still not trivial due to significant overlap of possible diameters as indicated by the box plots in Fig. 10 of the results section. Considering the box plot for the small class, it could also be seen that our assumption for using our reference size as the smallest possible diameter of a lymphoma nuclei could be considered valid, as this category does not significantly reach below the reference size.

Based on these findings, we used a brute force parameter testing method to test all possible combinations within a specified range of sensible values. These ranges are listed in Table 1. Ignoring possible duplicates, this method tested 178.364 combinations. Based on the known sizes of the labeled nuclei, each set was evaluated by its ability to separate the nuclei in the three classes (small, intermediate, large) so that the majority of measured nuclei represent the label for the whole image based on the expert’s consensus. Based on the comparison of the classification results done by the experts and the classification result using the individual parameter settings, we ranked the individual sets using scikit-learns’^32^ built-in F1 score method for multiple classes.

**Table 1:** The table presents the various parameter settings that were tested for the classification thresholds used in the study. The lower bound refers to the smallest value that was tested and was iteratively increased by the step size until the upper bound was reached. This testing process involved all possible combinations of parameter values within the specified lower and upper bound ranges, excluding any duplicates.

Parameter	Lower bound	Upper bound	Stepsize
Small lower limit	1.00	1.29	0.01
Small upper limit	1.18	1.24	0.01
Intermediate upper limit	1.35	1.51	0.01
Large upper limit	2.00	2.59	0.01

The best setting out of all tested parameter combinations was used for the classification part of our workflow.

### Workflow-generated output

**Figure 7:**
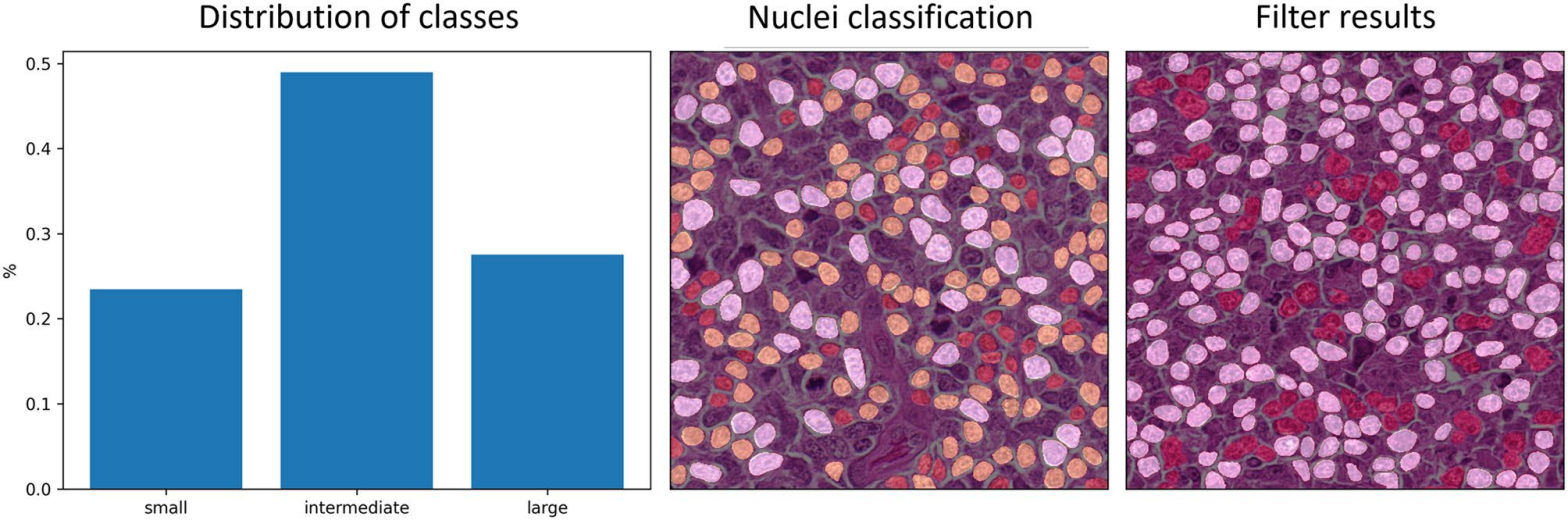
Example output combining all the mentioned methods of our workflow, represented by the class distribution on the left and the overlay of the classification of each nuclei (small: red, intermediate: yellow, large: white) on the original RGB image in the middle as well as a visual representation of the remaining nuclei (white) compared to the filtered objects (red) in the right image.

After the application of all the presented algorithms, our workflow not only provides a classification for the image based on the most common nuclei class but also provides an insight into the individual steps, as shown in Fig. 7. Despite the actual class distribution, which is used for the classification, our workflow provides overlays for the classification of each individual nuclei as well as the information on which objects were not included in the analysis due to their shape or size. Our workflow also provides the mentioned drop out rate of these invalid objects shown in the right image of Fig. 7.

## Results

In order to understand the abilities of our workflow’s segmentation and classification modules, we conducted separate testing and validation before combining them for overall test results. In the following subsection, we provide a detailed explanation of the individual test results.

### Segmentation and detection of lymphoma nuclei

**Figure 8:**
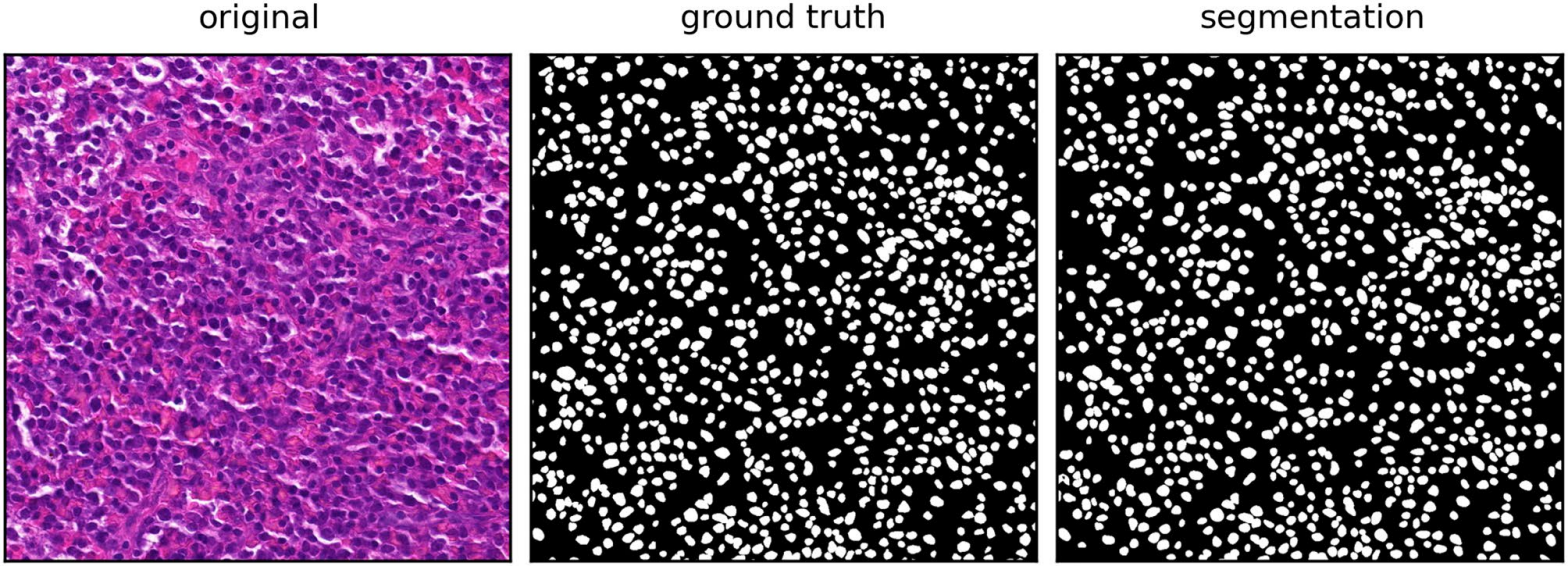
Visual representation of the segmentation result (right) provided by the Unet++ model using the raw RGB image (left) as input in comparison to the manually labeled ground truth mask (middle). On this test image, our model achieved an Dice score of 0.94.

**Figure 9:**
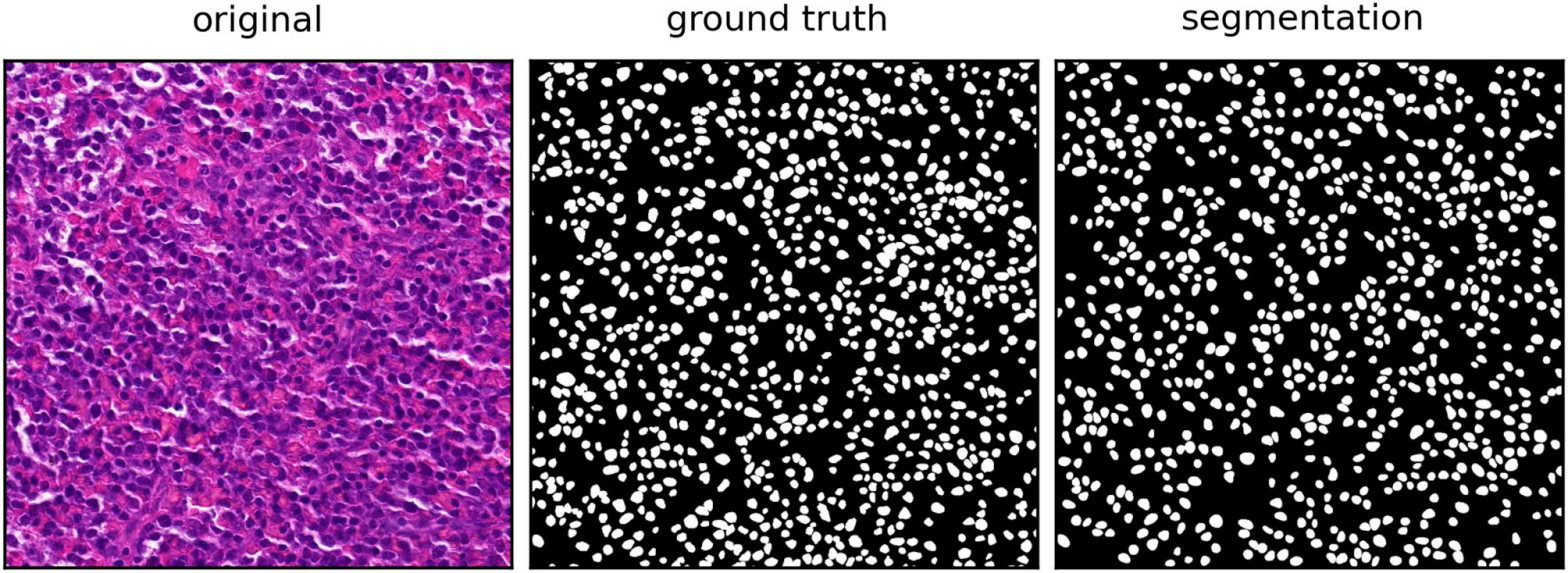
Visual representation of the segmentation result (right) provided by the Stardist model using the raw RGB image (left) as input in comparison to the manually labeled ground truth mask (middle). On this test image, our model achieved an Dice score of 0.74.

As depicted in Fig. 8, the segmentation outcome using the Unet++ represents a comparable binary mask for the used test image, in comparison to the ground truth mask, with a resulting overall Dice score for the nuclei segmentation quality of 0.8379 for all five test images. Based on the comparison with the individually labeled object within the ground truth image and the use of connected components labeling on the segmentation result, this combination achieved an object detection performance of F1 score = 0.8146. Despite the need for connected components labeling for the identification of the individual objects, the resulting detection score is even higher than the performance of the pre-trained and unmodified Stardist model on the exact same images (F1 = 0.7987), considering the quality measures in Table 2. The major downside of the used Stardist model is the comparably low binary segmentation accuracy with a Dice score of 0.7063 on the used test dataset. As shown in Fig. 9, this low score could be explained by the incomplete detection of nuclei visible in the mask but not segmented by the Stardist model. After analyzing the ground truth mask and the detection result, it became evident that some nuclei were not detected. However, the ones that were accurately detected were nearly identical to their corresponding counterparts in the ground truth.

**Table 2:** Comparative analysis of the performance metrics between Unet++ and Stardist for cell nuclei detection and segmentation.

Model	Object detection			Segmentation		
	Precision	Recall	F1	Dice	Sensitivity	Specificity
Unet++	0.9044	0.7411	0.8146	0.8379	0.8876	0.8764
Stardist	0.9360	0.6974	0.7993	0.7063	0.6289	0.9342

Although the binary segmentation of the pre-trained and unmodified Stardist model yielded a low Dice score, this model significantly reduced the necessity for filtering invalid objects, with only 5.76% of objects being excluded from the final classification, as opposed to the 23.11% exclusion rate when using the Unet++. This behavior can be explained by the high specificity of the binary segmentation mask produced by the Stardist model. These results reinforced our decision to employ these models for testing the image classification workflow, as they provide complementary properties: one being highly effective in detecting nearly all objects but with an increased need for filtering invalid objects, and the other having a higher rate of missed objects (low sensitivity) yet requiring less filtering.

### Classification parameter optimization

**Figure 10:**
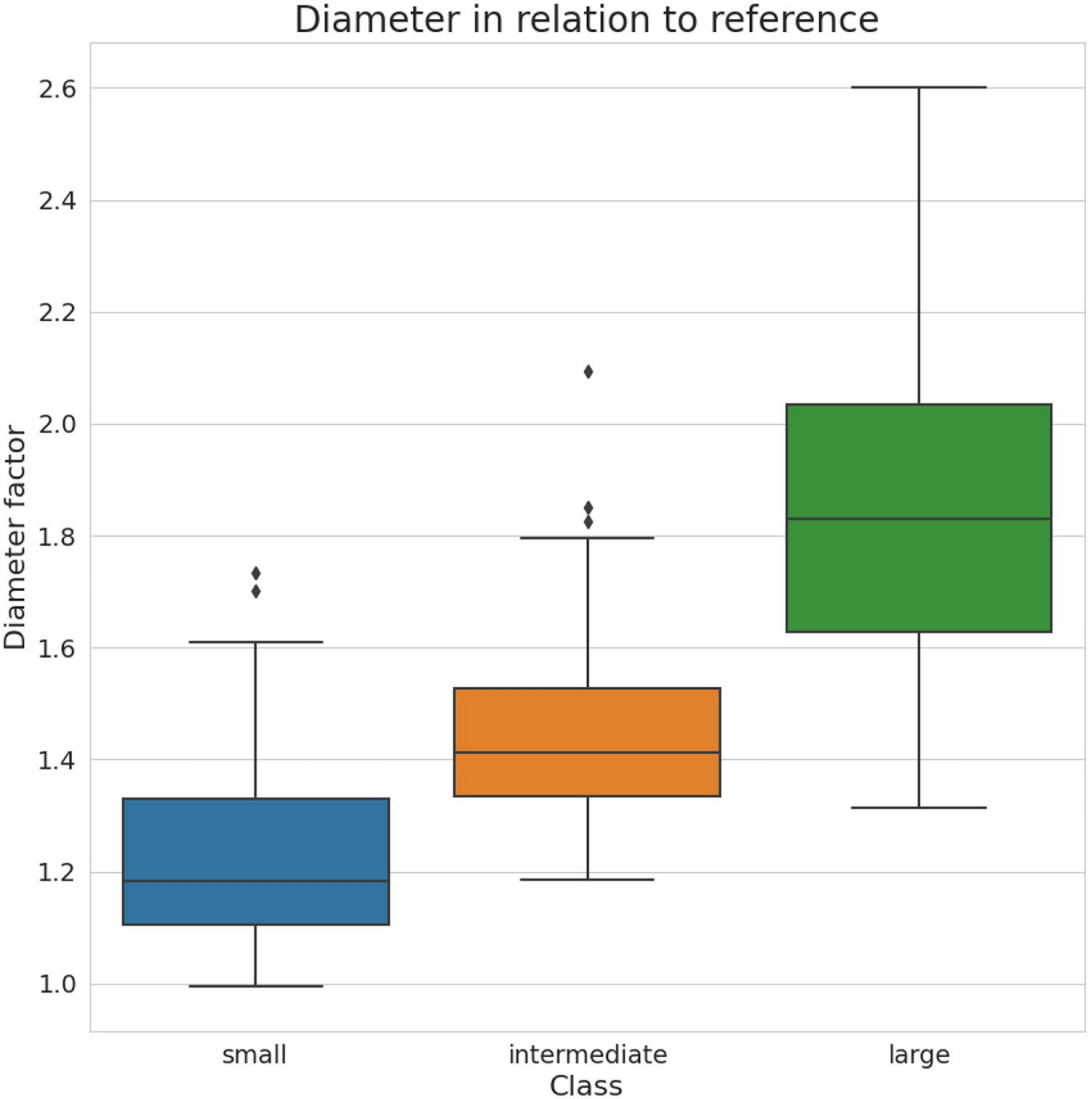
Boxplot representation of the three nuclear categories (small-sized, intermediate-sized, and large-sized) as classified independently by three pathologists. For each sample image, the three pathologists selected and measured 20 nuclei relevant to their classification decision. The boxplots indicate the diameter of these nuclei (totaling 60 per image) relative to our new reference.

To set a standard for our classification parameter tests, we used the measured value ranges of our optimization dataset, which are also visualized in the Boxplot of Fig. 10. Therefore, we used the lower limit of the small class and the upper range of the large nuclei as upper and lower thresholds. For the two thresholds in between, we used the mean of the upper quantile of the lower-sized class and the lower whisker of the upper-sized class. The resulting thresholds are listed in Table 3.

Using the thresholds of Table 3, our workflow achieved a majority classification F1 Score of 0.5860.

**Table 3:** Table of threshold based on the resulting values of the box plot shown in Fig. 10.

Class	Range of diameter values
Small	0.994969–1.258042 $\times$ reference
Intermediate	1.258042–1.420893 $\times$ reference
Large	1.420893–2.601033 $\times$ reference

Our optimized set of classification thresholds achieved a F1 Score of 0.8103 representing the best setting out of all tested parameter combinations listed in Table 4, which highlights the significant increase in classification quality compared to the F1 Score of only 0.5860 using the non-optimized parameters of Table 3.

**Table 4:** Table of classification thresholds based on the diameter of the individual nuclei compared to our measured reference diameter of small non-neoplastic lymphocytes. Each of the three classes is represented by an upper and lower limit represented by a multiple of our reference diameter.

Class	Range of diameter values
Small	1–1.21 $\times$ reference
Intermediate	1.21–1.5 $\times$ reference
Large	1.5–2.24 $\times$ reference

### Classification of whole images based on measured lymphoma nuclei

Next, we tested the overall classification accuracy of our whole workflow. The used test images were classified by three pathologists, representing the ground truth by majority vote (small, intermediate, large), subsequently referred to as majority classification.

**Table 5:** Confusion matrix of the canine test dataset depicting the classification performance of the algorithm based on the segmentation of the Unet++ model. The labels on the left indicate the class labels by pathologists’ majority classification, whereas the labels on the top represent the predicted labels by our workflow. The overall test accuracy was 92%.

	Small	Intermediate	Large
Small	5	0	0
Intermediate	1	7	1
Large	0	0	11

As represented by the confusion matrix in Table 5, the classification results on the test dataset indicate a strong performance in distinguishing between the three classes: small, intermediate, and large, with 92% correctly classified images compared to the pathologists’ majority classification. While the use of Unet++ leads to the accurate classification of most images, it provides minor misclassifications for the intermediate category.

**Table 6:** Confusion matrix of the canine test dataset, which shows the classification performance of three pathologists based on ten nuclei selected by each pathologist, which should represent the overall nuclear size predicted by the individual pathologist. The labels on the left indicate the class labels as defined by the consensus of our experts based on diagnostic experience. The mean accuracy of our pathologist was 74.22%.

	Small	Intermediate	Large	Small	Intermediate	Large	Small	Intermediate	Large
Small	4	0	0	5	0	0	4	1	0
Intermediate	2	5	2	2	6	1	1	3	5
Large	0	4	7	0	1	10	0	0	11
	Expert 1: 66.67%			Expert 2: 84%			Expert 3: 72%		

For comparing our workflow with results from pathologists, it is important to mention that our ground truth labels are not based on the ten nuclei selected by each pathologist but rather on the experience of the pathologists and defined rules for diagnosing the individual tumor samples. When comparing the accuracy of our workflow to the classification based on manually labeled nuclei by our experts (Table 6), we found that our workflow achieved a overall higher level of accuracy.

**Table 7:** Confusion matrix of the canine test dataset depicting the classification performance of the algorithm based on the segmentation of the pre-trained and unmodified Stardist model. The labels on the left indicate the class labels by pathologists’ majority classification, whereas the labels on the top represent the predicted labels by the model. The overall test accuracy was at 88%.

	Small	Intermediate	Large
Small	5	0	0
Intermediate	1	7	1
Large	0	1	10

As compared to the Unet++ results, the Stardist model leads to a similar classification performance with an accuracy of 88% (Table 7), despite being a pre-trained model without specific training on canine lymphoma. Differentiating between the three classes only shows some misclassifications for the intermediate category, similar to the Unet++-based algorithm.

### Transferability of image classification to feline lymphoma

Even though our classification thresholds were only optimized for canine lymphoma, we assumed that these parameters should also be applicable to feline lymphoma due to the similar size of their small non-neoplastic lymphocytes of around 4.17 µm (cat) compared to the 4.14 µm (dog). The application on feline data provides insight into the generalization ability of our workflow.

The approaches based on the Unet++ (Table 8) and on the Stardist (Table 9) models both resulted in high classification performance with a generally effective performance in differentiating between all three classes even though there are some misclassifications by differentiating the intermediate and large nuclei from each other. These results also show that the workflow based on the Stardist segmentation is able to provide a better classification accuracy compared to the one using the Unet. These results also reinforces our decision to use an already well-performing pre-trained and unmodified model and demonstrates that our workflow can be adapted for other nuclei segmentation neural networks.

**Table 8:** Confusion matrix for the classification performance using the Unet++ model (trained only with canine images) on the feline lymphoma images. The labels on the left indicate the class labels by pathologists’ majority classification, whereas the labels on the top represent the estimated labels by our workflow. The workflows’ accuracy on this dataset was 81.57%.

	Small	Intermediate	Large
Small	6	1	0
Intermediate	1	16	6
Large	0	0	9

**Table 9:** Confusion matrix for the classification performance using the pre-trained and unmodified Stardist model on the feline lymphoma images. The labels on the left indicate the class labels by pathologists’ majority classification, whereas the labels on the top represent the estimated labels by our workflow. The achieved accuracy on this dataset was 84.21%.

	Small	Intermediate	Large
Small	6	1	0
Intermediate	0	18	4
Large	0	1	8

**Table 10:** Confusion matrix that shows the individual classification performance based on the manually labeled nuclei of the three pathologists. The labels on the left indicate the class labels using the consensus of our experts, and the labels on the top of each block represent the prediction based on the pathologist’s nuclei measurements. The mean accuracy was 70.07%.

	Small	Intermediate	Large	Small	Intermediate	Large	Small	Intermediate	Large
Small	7	0	0	6	1	0	5	2	0
Intermediate	0	16	5	0	14	8	0	4	18
Large	0	0	9	0	0	9	0	0	9
	Expert 1: 86.49%			Expert 2: 76.32%			Expert 3: 47.39%		

As with the canine test data, we also analyzed the classification accuracy using the experts’ estimates for the feline test images. As indicated by the results in Table 10, pathologists had a significantly lower classification accuracy of 70.07% compared to our workflows’ accuracy of 81.57 and 84.21%.

**Figure 11:**
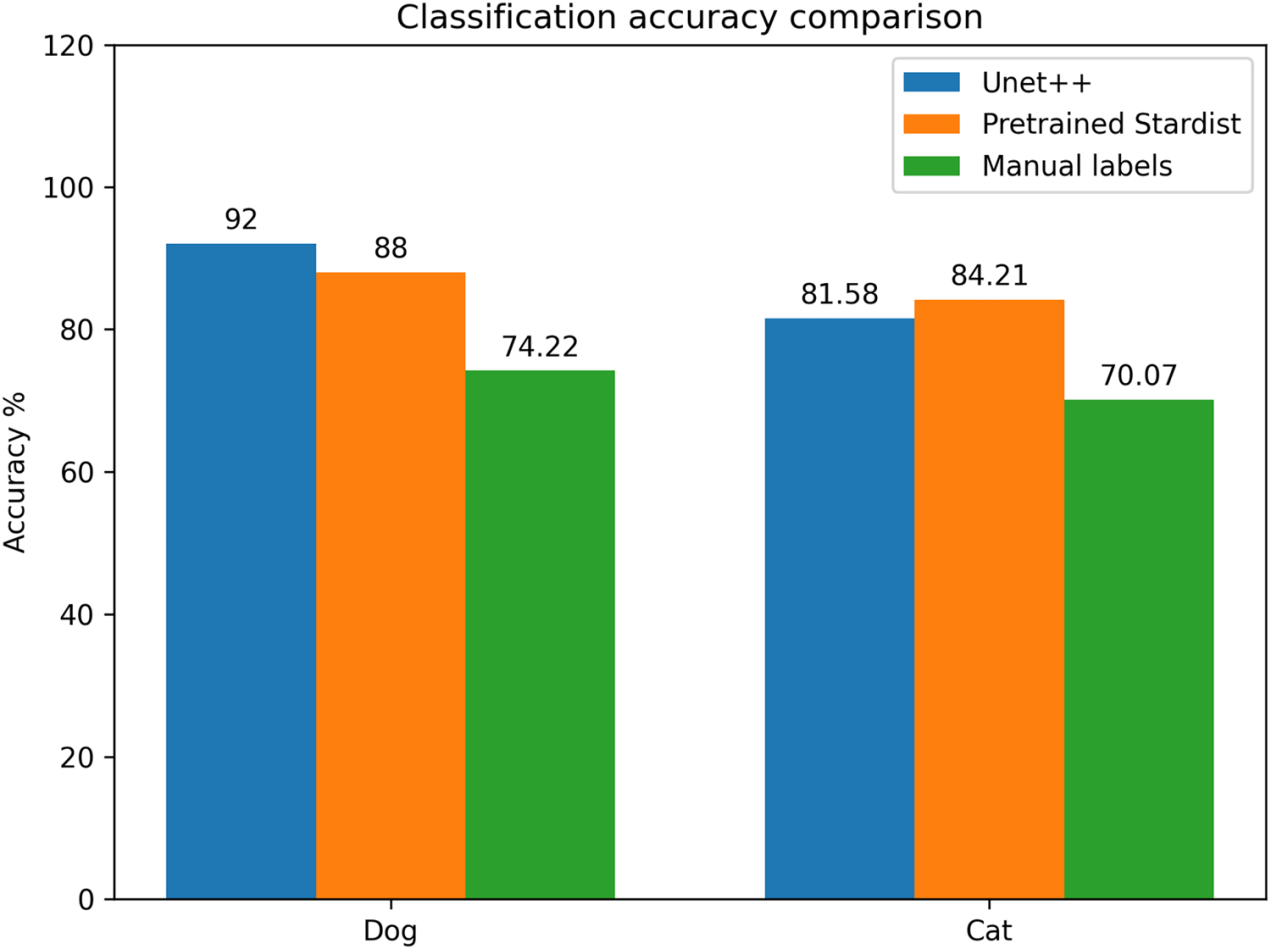
Bar chart comparison of the achieved lymphoma subtype classification accuracy using the mentioned methods for providing the segmented nuclei within the histological images.

The comparison of the achieved classification accuracies in Fig. 11 shows nearly indistinguishable results using the Unet++ and the pre-trained and unmodified Stardist model. The results on both datasets show that our manually labeled nuclei dataset led to a worse classification than the deep learning-supported methods.

## Discussion

Our study results demonstrate the potential of our automated workflow in distinguishing between three nuclei categories (small, intermediate, and large) of lymphomas in dogs and cats. Moreover, the workflow offers valuable insights into the classification process and provides information about the nuclear size distribution. As our results on the routine evaluation by pathologists indicate, obtaining reproducible classification for identical slides through individual estimations is difficult. Our workflow fulfills this need for increased reproducibility through automated measurement and classification of the individual nuclei. During the classification tests, our system processed 25 images with dimensions of 1024 $\times$ 1024 pixels per minute, utilizing a Nvidia Titan RTX graphics card, which would allow a routine integration into a diagnostic workflow. However, these images represent a small proportion of the entire whole slide image and, thus, our current workflow still relies on manual selection of regions of interest by pathologists. While this hinders the fully automated processing of lymphoma cases, it also ensures that an appropriate tumor region is selected and markedly decreases computational costs. For classification of the mean nuclear size, analysis of entire whole slide images would probably not be beneficial, providing that the nuclear size is similar in all tumor regions. Future research may focus on automated selection of an appropriate tumor region.

Using the publicly available pre-trained and unmodified Stardist model, we have successfully demonstrated that our workflow provides the flexibility for the adaptation to other types of segmentation models, providing a segmentation mask for the nuclei. The used Stardist model was not fine-tuned on our lymphoma data, which should highlight the capability of our classification and filtering workflow, still providing a high level of accuracy based on the segmentation of this neural network. Despite the initial lack of interpretability due to the use of a Unet++ or the pre-trained Stardist model (both considered as black-box models), our workflow was still able to provide detailed insight into the classification of the slide images based on the provided segmentation mask, including the distribution of the detected nuclei classes, the amount and reason of excluded objects, as well as the class and area for each detected object. Providing insight into the classification process, independent of the neural network used to supply the segmentation mask, represents a major benefit of our classification workflow. These results highlight the potential for broader applications in tumor diagnostics and the possibility of further refining the workflow to process other types of human and animal tumors based on the presented concept of separating the image segmentation and feature-based classification. Our workflow allows a fast and reproducible cell nuclei classification and analysis of the predominance class. It, therefore, represents a useful tool to support clinical decisions but, at this point, should not be considered a stand-alone diagnostic tool.

A limitation of our study is the amount of available data due to the need for expert labels and individually marked nuclei within the images. This limitation led us to the decision to include our data sourced from cats only within the test dataset, which has provided insight into the generalization performance of our workflow. The results have demonstrated that the workflow provides a suitable classification even if data from this species was not included in the modeling and parameter optimization. However, it cannot be confirmed that this applies to other species without repeating the modeling and optimizing parameters for the classification module.

Our goal was to provide a workflow for analyzing and classifying histological images of canine lymphoma based on the segmentation of the neoplastic nuclei. While, we consider the novelty of our work to be the post-processing of the provided segmentation mask for obtaining best classification results, a well-performing segmentation model is the foundation for this work. Therefore, it was outside the scope of this work to compare the segmentation quality of several neural network architectures. Nevertheless, we evaluated two state-of-the art neural networks in our study based on previously published architectures. The fist model, a Unet++, was specifically trained on our data and served as the baseline. The second model, a publically available pre-trained Stardist model, was not optimized for our data and was used to demonstrate two things: 1) instance segmentation networks can be used instead of a semantic segmentation network, and 2) networks like Stardist can still be used, even if the nuclear segmentation is not specific for the cell type of interest, when combined with our proposed filter mechanism and classification module.

This selection of segmentation models should emphasize that our workflow is intended to work with different kinds of segmentation neural networks due to the separation of segmentation and classification. It would also be possible to use a classification neural network using the overall images class as input and the final classification label as output. This end-to-end classification of the images would, however, not fulfill our goals, which were to provide as much insight into the classification process as possible and thereby gain the users’ trust. This requirement was fulfilled by combining a segmentation neural networks with image processing. Even if our segmentation neural networks are considered black-box models, their provided segmentation masks are more understandable by the users than the confidence values of classification neural networks.

## Data Availability

All of our datasets are publically available through the following link: https://git.fh-ooe.at/fe-extern/Lymphoma-Dataset.git.
